# Patient experience of CT colonography and colonoscopy after fecal occult blood test in a national screening programme

**DOI:** 10.1007/s00330-016-4428-x

**Published:** 2016-06-10

**Authors:** Andrew A. Plumb, Alex Ghanouni, Colin J. Rees, Paul Hewitson, Claire Nickerson, Suzanne Wright, Stuart A. Taylor, Steve Halligan, Christian von Wagner

**Affiliations:** 1 Centre for Medical Imaging, Division of Medicine, University College London, 3rd Floor East, 250 Euston Road, London, NW1 2PG UK; 2 Health Behaviour Research Centre, Department of Epidemiology and Public Health, University College London, London, UK; 3 Durham University School of Medicine, Pharmacy and Health, Durham, UK; 4 Health Services Research Unit, Nuffield Department of Population Health, University of Oxford, Oxford, UK; 5 NHS Cancer Screening Programmes, Fulwood House, Sheffield, UK

**Keywords:** Colon cancer, Computed tomography, Endoscopy, Fecal occult blood test, CT colonography (CTC)

## Abstract

**Objective:**

To investigate patient experience of CT colonography (CTC) and colonoscopy in a national screening programme.

**Methods:**

Retrospective analysis of patient experience postal questionnaires. We included screenees from a fecal occult blood test (FOBt) based screening programme, where CTC was performed when colonoscopy was incomplete or deemed unsuitable. We analyzed questionnaire responses concerning communication of test risks, test-related discomfort and post-test pain, as well as complications. CTC and colonoscopy responses were compared using multilevel logistic regression.

**Results:**

Of 67,114 subjects identified, 52,805 (79 %) responded. Understanding of test risks was lower for CTC (1712/1970 = 86.9 %) than colonoscopy (48783/50975 = 95.7 %, *p* < 0.0001). Overall, a slightly greater proportion of screenees found CTC unexpectedly uncomfortable (506/1970 = 25.7 %) than colonoscopy (10,705/50,975 = 21.0 %, *p* < 0.0001). CTC was tolerated well as a completion procedure for failed colonoscopy (unexpected discomfort; CTC = 26.3 %: colonoscopy = 57.0 %, *p* < 0.001). Post-procedural pain was equally common (CTC: 288/1970,14.6 %, colonoscopy: 7544/50,975,14.8 %; *p* = 0.55). Adverse event rates were similar in both groups (CTC: 20/2947 = 1.2 %; colonoscopy: 683/64,312 = 1.1 %), but generally less serious with CTC.

**Conclusions:**

Even though CTC was reserved for individuals either unsuitable for or unable to complete colonoscopy, we found only small differences in test-related discomfort. CTC was well tolerated as a completion procedure and was extremely safe. CTC can be delivered across a national screening programme with high patient satisfaction.

***Key Points*:**

• *High patient satisfaction at CTC is deliverable across a national screening programme.*

• *Patients who cannot tolerate screening colonoscopy are likely to find CTC acceptable.*

• *CTC is extremely safe; complications are rare and almost never serious.*

• *Patients may require more detailed information regarding the expected discomfort of CTC.*

## Introduction

Patient experience is fundamental to high-quality healthcare, and is important to patients [1] and policy-makers [2]. Although healthcare services are increasingly scrutinized, many quality assessments overlook patient experience. This is particularly important for screening programmes, as they are predicated on high uptake and acceptability. Mass screening for colorectal cancer (CRC) has been introduced in many countries [3], most often using fecal occult blood testing (FOBt) followed by colonoscopy for those testing positive [4, 5]. This reduces CRC-related mortality, proven by randomized trials [6].

In a proportion of individuals, it is not possible to intubate the entire colon–whether due to frailty, technical difficulty, or simply refusal. In this situation, one alternative is to offer CT colonography (CTC), since it is highly sensitive for CRC and polyps both as a primary screening tool [7, 8] and in FOBt-positive individuals [9]. Pan-European consensus recommends CTC when colonoscopy is incomplete or not possible [10–12]. Relatively little is known regarding the relative acceptability of CTC versus colonoscopy in this setting.

CTC is generally well-tolerated in comparison to colonoscopy [13–15]. Two randomized studies, one of asymptomatic screenees [16] and one of symptomatic older patients [17] reported similar acceptability of CTC and colonoscopy, with the balance marginally in favour of colonoscopy for screenees [18] and CTC for symptomatic patients [19]. However, it is unknown whether these findings are replicated in routine clinical practice beyond randomized trials. Furthermore, the relevance of these findings to FOBt-based programmes, in which direct colonic investigation is a second-stage test, is also unknown.

Here we describe patient experience of CT colonography in a national FOBt-based CRC screening programme when colonoscopy is not possible or incomplete.

## Methods

### Ethical permissions

Permission to access anonymized, routinely-coded data was granted by the English Bowel Cancer Screening Programme. Approval for evaluation of retrospective, anonymized data was granted by our institutional research office.

### Participants

The English Bowel Cancer Screening Programme (BCSP) is a national screening programme using biennial FOBt and colonoscopy for those testing positive [20]. Eligible residents aged 60-74 years are mailed a FOBt kit, and those testing positive are invited for a clinical assessment and colonoscopy at a local hospital “screening centre”. CTC is substituted if colonoscopy is judged inappropriate or infeasible (e.g. due to co-morbidity) or if colonoscopy is incomplete (e.g. impassable tumour/stricture, technical failure). All patients are assessed by a trained healthcare professional prior to colonoscopy or CTC, and the decision to refer to CTC rather than colonoscopy due to unsuitability for the latter is ratified by a screening-accredited colonoscopist. All participants undergoing a colonic test are sent a standard questionnaire 30 days after the process. Here we present data for screenees tested between January 1st, 2011, and December 31st, 2012, (the first two full calendar years after programme roll-out) and focus on CTC-related outcomes; colonoscopy-specific data will be reported elsewhere.

### Procedures

CTC requirements include dual patient positioning, fecal tagging, carbon dioxide insufflation, multislice scanners, trained staff, and a CTC workstation. There are no stipulations regarding use or quantity of purgation; recent survey data suggest the commonest strategy is to use either two sachets of sodium picosulfate/magnesium citrate (Picolax, Ferring Pharmaceuticals, West Drayton, UK) or 100-150 mL of sodium diatrizoate/meglumine diatrizoate (Gastrografin, Bayer plc, Newbury, UK) as a combined purgation/tagging agent. Use of intravenous contrast is discouraged unless specifically needed (e.g. for tumour staging). Radiologists must interpret >100 cases per annum (both screening and symptomatic) before commencing work for the programme [12]. Sixty percent of centres perform >300 CTC per year [21]. All programme colonoscopies are performed by screening-accredited colonoscopists, a process requiring (a) completion of >1000 colonoscopies, (b) success in a written multiple-choice question examination, and (c) successful evaluation of two observed colonoscopies by an external assessor [22]. Sedoanalgesia is administered as required.

### Outcome measures

The patient questionnaire addresses the screening invitation, the pre-colonic test assessment, the preparation for their colonic test, the test itself (including discomfort), post-test symptoms, communication of results, and privacy/dignity. Most items are answered with a five-point Likert-type scale (“strongly agree” to “strongly disagree”) or a binary “yes/no” response.

### Data recording and extraction

We used the BCSP database to extract: participant age, sex, date of screening, screening centre used, screening round (i.e. number of times previously screened via FOBt), colonic test performed, test result, need for any subsequent tests (e.g. colonoscopy following a positive CTC result), recorded complications (entered by screening programme staff), and questionnaire results. Socioeconomic deprivation was estimated using the index of multiple deprivation (IMD) from 2007, a composite deprivation measure linked to postal (ZIP) code. Complications for CTC were divided into direct (i.e. attributed to CTC) and downstream (i.e. after an additional test following CTC).

### Data analysis

Data were coded using SPSS v21 for Windows (IBM Corp, Armonk, NY, USA) and analyzed with R version 3.0.1 for Mac (R Foundation for Statistical Computing, Austria, Vienna). Participants not responding to any questionnaire item were excluded from the patient experience analysis but were included when calculating complication rates. Missing questionnaire data for those who responded to at least one questionnaire item were handled via 10 multiple imputations using the mice package for R under the missing at random assumption [23]. Variables used in the imputation model were patient age, sex, socio-economic deprivation, screening round, test used, screening outcome, and questionnaire results.

Following imputation, questionnaire data with a Likert-type scale were collapsed into binary categories of “agreement” and “non-agreement” because initial analysis showed the data violated the proportional odds assumption. Subsequently, multilevel binary logistic regression was used, with the diagnostic test being performed (i.e. CTC or colonoscopy) as the explanatory variable. Covariates in the regression model were the same as those used for the imputation model, although the occurrence of complications and other questionnaire responses were excluded. The model accounted for clustering of screenees into screening centres. All statistical tests were conducted on each multiply imputed data set and pooled according to Rubin [24].

Since we expected that screenees undergoing CTC following colonoscopy (i.e. incomplete colonoscopy) and those triaged directly for CTC (i.e. judged unsuitable for colonoscopy) may have had differing experience, we compared these subgroups. Additionally, we expected that those who underwent colonoscopy following CTC were more likely to require polypectomy (contingent on a CTC abnormality), which increases procedural complexity; hence, subgroup analysis was also performed here.

CTC questionnaire results for individual centres were compared using funnel plots constructed with exact binomial control limits at 2 and 3 standard deviations [25]. We also used logistic regression to examine whether centres using non-laxative preparation for CTC (data taken from reference [21]) had differences in test discomfort.

## Results

**Fig. 1:**
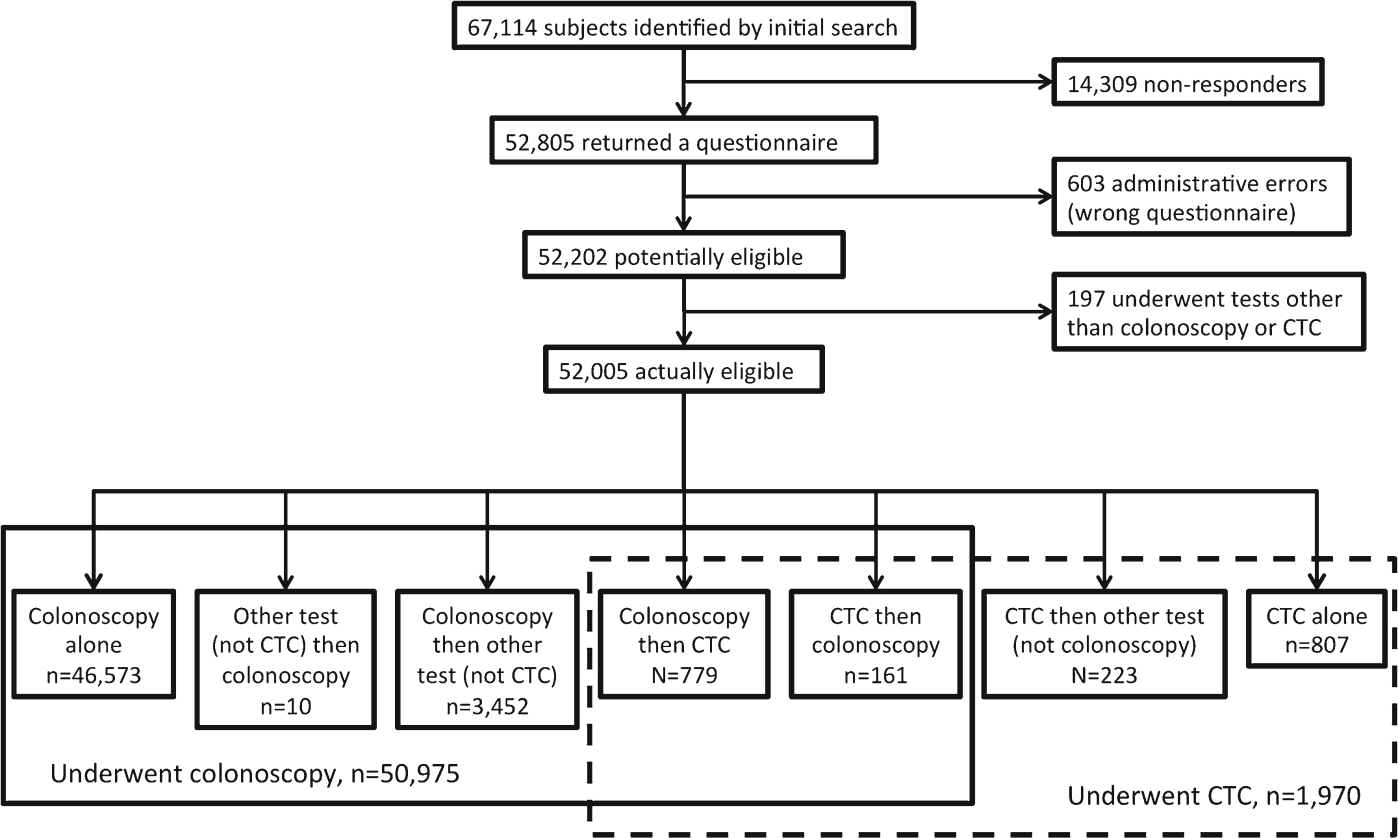
Study flowchart of inclusions and exclusions (also see text). The term “other test” refers to flexible sigmoidoscopy, barium enema, and non-colonographic abdominopelvic CT scanning, all of which were occasionally used in the programme during the study period (the latter two tests have since been removed as testing options)

Of 67,114 potentially eligible screenees, 52,805 returned a questionnaire (78.7 % overall; 51,554/65,197 = 79.1 % for colonoscopy; 2018/2946 = 68.5 % for CTC; Fig. 1). Of these, 603 were excluded due to administrative or data entry errors (incorrect questionnaire mailed, or questionnaire date before test date). One hundred and ninety-seven individuals had no record of undergoing either CTC or colonoscopy. Ultimately, 52,202 questionnaires were analysed, 50,975 for colonoscopy and 1,970 for CTC (Fig. 1).

Demographics and screening characteristics are summarised in Table 1. Slightly more respondents (1191/1970 = 60.0 %) underwent CTC because of contraindications to colonoscopy rather than incomplete colonoscopy (779/1970, 40.0 %). Individuals who underwent CTC were older (CTC: mean 66.9 years; colonoscopy: mean 66.3 years, *p* < 0.0001) and more likely to be female (CTC: 1017/1970 = 51.6 % female; Colonoscopy: 21,111/50975 = 41.4 %, *p* < 0.0001, Table 1). Average socio-economic deprivation was greater for those who underwent CTC (median deprivation = 48th percentile) than colonoscopy (median deprivation = 42nd percentile), with no difference between the CTC subgroups.

**Table 1:** Demographics of screenees undergoing CTC and optical colonoscopy (OC). Deprivation quartiles are derived from the entire English national dataset. Between group comparative p values are for all CTC screenees vs. all colonoscopy screenees (far right) and for the CTC subgroups (middle column)

Variable	Test performed					*p* value (CTC vs. OC)
	CTC (n = 1970)				OC (n = 50,975)	
	Unsuitable for OC (n = 1191)	Incomplete OC (n = 779)	Both groups (n = 1970)	*p* value (between CTC subgroups)		
Female patients	552 (46.3 %)	465 (59.7 %)	1017 (51.6 %)	<0.001	21,111 (41.4 %)	<0.001
Age in years (median, IQR)	66 (63 to 70)	66 (63 to 69)	66 (63 to 69)	0.08	66 (63 to 69)	<0.001
Deprivation score						
Lowest quartile (least deprived)	265 (22.2 %)	200 (25.7 %)	465 (23.6 %)	0.09	15,000 (29.4 %)	<0.001
2nd quartile	334 (28.0 %)	213 (27.3 %)	547 (27.8 %)	0.77	14,569 (28.6 %)	0.45
3rd quartile	305 (25.6 %)	215 (27.6 %)	520 (26.4 %)	0.35	12,388 (24.3 %)	0.036
Highest quartile (most deprived)	283 (23.8 %)	151 (19.4 %)	434 (22.0 %)	0.03	8925 (17.5 %)	<0.001
Missing	4 (0.34 %)	0 (0.0 %)	4 (0.20 %)	0.16	93 (0.18 %)	0.78
Year screened						
2011	691 (58.0 %)	482 (61.9 %)	1173 (59.5 %)	-	29595 (58.1 %)	-
2012	500 (42.0 %)	297 (38.1 %)	797 (40.5 %)	0.10	21380 (41.9 %)	0.20
Screening round						
Prevalent (individual’s first screening)	527 (44.2 %)	358 (46.0 %)	885 (44.9 %)	-	22159 (43.5 %)	-
Incident (subsequent screening of that individual)	664 (55.8 %)	421 (554.0 %)	1085 (55.1 %)	0.48	28816 (56.5 %)	0.21

Relatively few responses were missing, ranging from 0.6-15.6 % for the different questionnaire items. There were no differences between complete-case analysis and multiply imputed analysis for any items; multiply imputed responses are reported here, with complete-case data shown in Table 2.

**Table 2:** Comparison between responses for CTC and optical colonoscopy (OC). For each questionnaire item, the number (and percentage) answering either “strongly agree” or “agree” is presented, split by test modality, for both multiple imputation and complete case analysis. Items marked by an asterisk required binary (yes/no) responses. For complete case analysis, the number of individuals responding to each questionnaire item is shown as the denominator. For multiply imputed analyses, numerators and denominators are averages across all imputations. Odds ratios, 95 % confidence intervals and associated *p* values are derived from the logistic regression model for each item. The odds ratio is the odds of agreement with a given question being greater for OC than CTC

Item	Complete cases				Multiple imputation			
	Number agreeing (percentage)		Odds ratio (95 % CI)	*p* value	Number agreeing (percentage)		Odds ratio (95 % CI)	*p* value
	CTC (%)	OC (%)			CTC (%)	OC (%)		
Items regarding the pre-test experience								
I found the test kit easy to use	1752/1958 (89.5 %)	46,055/50,750 (90.7 %)	1.12 (0.96-1.30)	0.21	1765/1970 (89.6 %)	46,285/50,975 (90.8 %)	1.14 (0.98-1.33)	0.09
I understood the risks of having the colonoscopy/radiology test	1561/1786 (87.4 %)	47,811/50,007 (95.6 %)	3.12 (3.01-3.19)	<0.0001	1712/1970 (86.9 %)	48,783/50,975 (95.7 %)	3.01 (2.59-3.51)	<0.0001
I understood the benefits of having the colonoscopy/radiology test	1677/1786 (93.9 %)	49,126/50,033 (98.2 %)	4.09 (3.94-4.24)	<0.0001	1844/1970 (93.6 %)	50,057/50,975 (98.2 %)	3.31 (2.61-4.19)	<0.0001
I was given clear information on how to take bowel prep medicine (laxative)	1824/1913 (95.3 %)	49,069/50,135 (97.9 %)	1.55 (1.44-1.65)	<0.0001	1875/1970 (95.2 %)	49,905/50,975 (97.9 %)	1.89 (1.50-2.40)	<0.0001
I signed the consent form before entering the room*	1363/1917 (71.1 %)	46,370/50,369 (92.1 %)	4.74 (4.25-5.29)	<0.0001	1365/1970 (69.3 %)	47,050/50,975 (92.3 %)	4.69 (4.19-5.28)	<0.0001
Items regarding the test procedure itself								
The colonoscopy/radiology test was more uncomfortable than I expected	425/1695 (25.1 %)	10,253/50,091 (20.5 %)	0.77 (0.70-0.84)	<0.0001	506/1970 (25.7 %)	10,705/50,975 (21.0 %)	0.81 (0.72-0.91)	<0.0001
I asked for the colonoscopy/radiology test to be stopped or paused*	94/1694 (5.5 %)	2409/50127 (4.8 %)	0.86 (0.69-1.07)	0.17	114/1970 (5.8 %)	2600/50,975 (5.1 %)	0.90 (0.70-1.11)	0.28
I feel that my privacy was maintained as much as possible during my visit to hospital	1726/1811 (95.3 %)	49,016/50,050 (97.9 %)	2.26 (2.13-2.40)	<0.0001	1879/1970 (95.4 %)	49,905/50,975 (97.9 %)	2.30 (1.82-2.91)	<0.0001
I feel I was treated with respect during my visit to hospital	1743/1813 (96.1 %)	49,190/49,981 (98.4 %)	2.17 (2.01-2.33)	<0.0001	1895/1970 (96.2 %)	50,159/50,975 (98.4 %)	2.27 (1.75-2.87)	<0.0001
Items regarding the post-test experience								
After the colonoscopy/radiology test, I suffered with pain in my bottom and/or stomach*	246/1666 (14.8 %)	7235/49,714 (14.6)	0.96 (0.83-1.11)	0.61	288/1970 (14.6 %)	7544/50,975 (14.8 %)	1.01 (0.83-1.16)	0.77
The screening practitioner rang me within 7 days, to discuss my results*	1307/1645 (79.5 %)	39,432/47,691 (82.7 %)	1.29 (1.14-1.47)	<0.0001	1564/1970 (79.4 %)	42,105/50,975 (82.6 %)	1.24 (1.12-1.47)	<0.0001
I understood what my colonoscopy/radiology results meant	1511/1663 (90.9 %)	47,819/49,367 (96.9 %)	3.17 (3.01-3.30)	<0.0001	1783/1970 (90.5 %)	49,395/50,975 (96.9 %)	3.26 (2.71-3.91)	<0.0001

### Items regarding pre-test experience

Satisfaction with the communication of risks and benefits of CTC was high. Respondents agreed or strongly agreed they understood both risks (1712/1970 = 86.9 %) and benefits (1844/1970 = 93.6 %) of CTC. Understanding was slightly higher for colonoscopy, with 48,783/50,975 = 95.7 % agreeing they had understood its risks (odds ratio [OR] = 3.01, 95 %CI 2.59-3.51, *p* < 0.0001) and 50,057/50,975 = 98.2 % understanding benefits (OR = 3.31, 95 %CI 2.61-4.19, *p* < 0.0001). Respondents found bowel preparation instructions clear, for both CTC (1875/1970 = 95.2 % agreement) and colonoscopy (49,905/50,975 = 97.9 %), with a small, but statistically significant difference in favour of colonoscopy (OR = 1.89, 95 %CI 1.50-2.40, *p* < 0.0001, Table 2).

### Items regarding the test procedure

Approximately one quarter (506/1970 = 25.7 %) of respondents found CTC more uncomfortable than expected. This was a larger proportion than for colonoscopy (10,705/50,975 = 21.0 %; OR = 0.81, 95 %CI 0.72-0.91, *p* < 0.0001). This difference remained even for patients who denied receiving sedation during their colonoscopy (Table 3). There was no significant difference in asking for the test to be stopped/paused (CTC: 114/1970 = 5.8 %; colonoscopy: 2600/50,975 = 5.1 %, *p* = 0.28), whether or not patients reported receiving sedation for their colonoscopy (Table 3). Almost all individuals agreed they had been treated with both privacy and respect for both tests, although again there were small, but statistically significant differences in favour of colonoscopy (Table 2).

**Table 3:** Comparison between responses for discomfort-related variables at CTC and optical colonoscopy (OC), split by self-reported administration of sedation at colonoscopy. All data presented are derived from complete cases only; no differences were observed for the multiply imputed data (not shown). Minor differences in denominators for each questionnaire item and from Table 2 are due to incomplete responses from some individuals. Odds ratios (OR), 95 % confidence intervals (95 %CI) and associated *p* values are derived from the logistic regression model for each item. Higher ORs indicate respondents were more likely to agree with that questionnaire item for the first named test vs. the second

Item	Number agreeing (percentage)			Unsedated OC (vs. sedated OC)		CTC (vs. sedated OC)		CTC (vs. unsedated OC)	
	OC (%)		CTC (%)	OR (95%CI)	*p* value	OR (95%CI)	*p* value	OR (95%CI)	*p* value
Questionnaire item	Sedated	Unsedated							
The colonoscopy/radiology test was more uncomfortable than I expected	7995/39,429 (20.3 %)	2092/9193 (22.8 %)	425/1695 (25.1 %)	1.21 (1.14 – 1.28)	<0.0001	1.30 (1.21 – 1.39)	<0.0001	1.15 (1.04 – 1.26)	0.011
I asked for the colonoscopy/radiology test to be stopped or paused	1867/39,441 (4.7 %)	587/9195 (6.4 %)	94/1694 (5.5 %)	1.38 (1.21 – 1.55)	<0.0001	1.15 (0.97 – 1.32)	0.22	0.87 (0.72 – 1.03)	0.12
After the colonoscopy/radiology test, I suffered with pain in my bottom and/or stomach	5757 / 38,856 (14.8 %)	1264/9056 (14.0 %)	246/1666 (14.8 %)	0.95 (0.78 – 1.11)	0.59	1.00 (0.80 – 1.20)	0.99	0.94 (0.75 – 1.13)	0.69

### Items regarding the post-test experience

There was no significant difference in the proportion of respondents who recalled suffering rectal/abdominal pain following their diagnostic test (CTC: 288/1970,14.6 %, colonoscopy: 7544/50,975,14.8 %; *p* = 0.55). Again, there was no discernable effect of sedation on this questionnaire item (Table 3). Those who had undergone CTC were less likely to have received their results within seven days (1564/1970 = 79.4 %) than for colonoscopy (42,105/50,975 = 82.6 %; *p* < 0.0001). A greater proportion of those who underwent colonoscopy agreed they understood their results (49,395/50,975 = 96.9 %) than for CTC (1783/1970 = 90.5 %; OR = 3.26, 95 %CI 2.71-3.91, *p* < 0.0001; Table 2).

### Variation by indication for CTC

There were few differences between questionnaire results for the two CTC subgroups (Table 4). Only the assessment of clarity of bowel preparation and signing of informed consent were significantly different, both judged superior for the group with prior incomplete colonoscopy. Specifically, unexpected discomfort and post-test anorectal/abdominal pain were no different between the two CTC subgroups.

**Table 4:** Comparison between CTC subgroups. For each questionnaire item, the number (and percentage) answering either “strongly agree” or “agree” is presented, split by the indication for CTC, for both multiple imputation and complete case analysis. Items marked by an asterisk required binary (yes/no) responses. For complete case analysis, the number of individuals responding to each questionnaire item is shown as the denominator. For multiply imputed analyses, numerators and denominators are averages across all imputations. Odds ratios, 95 % confidence intervals and associated *p* values are derived from the logistic regression model for each item. The odds ratio is the odds of agreement with a given question being greater for patients having CTC after incomplete optical colonoscopy (OC) rather than because they had been judged unsuitable for OC

Item	Complete cases				Multiple imputation			
	Number (percentage) agreeing		Odds ratio (95 % CI)	*p* value	Number (percentage) agreeing		Odds ratio (95 % CI)	*p* value
	Unsuitable for OC (%)	Incomplete OC (%)			Unsuitable for OC (%)	Incomplete OC (%)		
Items regarding the pre-test experience								
I found the test kit easy to use	1059/1183 (89.5 %)	693/775 (89.4 %)	0.96 (0.70-1.31)	0.79	1066/1191 (89.5 %)	697/779 (89.4 %)	0.96 (0.69-1.32)	0.88
I understood the risks of having the radiology test	969/1105 (87.7 %)	592/681 (86.9 %)	0.90 (0.66-1.23)	0.51	1042/1191 (87.5 %)	675/779 (86.6 %)	0.91 (0.65-1.25)	0.54
I understood the benefits of having the radiology test	1046/1113 (94.0 %)	631/673 (93.7 %)	0.91 (0.59-1.41)	0.67	1117/1191 (93.8 %)	729/779 (93.6 %)	0.95 (0.61-1.45)	0.79
I was given clear information on how to take bowel prep medicine (laxative)	1083/1150 (94.2 %)	741/763 (97.1 %)	1.84 (1.09-3.08)	0.02	1123/1191 (94.2 %)	756/779 (97.1 %)	1.84 (1.08-3.10)	0.03
I signed the consent form before entering the room*	699/1090 (64.1 %)	469/638 (73.5 %)	0.60 (0.48-0.75)	<0.001	781/1191 (65.6 %)	574/779 (73.7 %)	0.71 (0.55-0.88)	0.005
Items regarding the test procedure itself								
The radiology test was more uncomfortable than I expected	263/1061 (24.8 %)	162/634 (25.5 %)	1.08 (0.85-1.38)	0.52	299/1191 (25.1 %)	205/779 (26.3 %)	1.07 (0.84-1.37)	0.54
I asked for the radiology test to be stopped or paused*	51/1056 (4.8 %)	43/638 (6.7 %)	1.02 (0.61-1.70)	0.35	58/1191 (4.9 %)	54/779 (6.9 %)	0.97 (0.59-1.60)	0.40
I feel that my privacy was maintained as much as possible during my visit to hospital	1052/1114 (94.4 %)	674/697 (96.7 %)	1.54 (0.90-2.63)	0.12	1126/1191 (94.5 %)	752/779 (96.5 %)	1.44 (0.87-2.40)	0.15
I feel I was treated with respect during my visit to hospital	1069/1117 (95.7 %)	674/696 (96.8 %)	1.25 (0.72-2.15)	0.42	1140/1191 (95.7 %)	752/779 (96.6 %)	1.15 (0.67-2.00)	0.45
Items regarding the post-test experience								
After the radiology test, I suffered with pain in my bottom and/or stomach*	162/1048 (15.5 %)	84/618 (13.6 %)	1.19 (0.87-1.62)	0.33	187/1191 (15.7 %)	108/779 (13.9 %)	1.23 (0.90-1.66)	0.17
The screening practitioner rang me within 7 days, to discuss my results*	810/1039 (78.0 %)	497/606 (82.0 %)	0.96 (0.72-1.28)	0.26	928/1191 (77.9 %)	633/779 (81.3 %)	0.97 (0.73-1.29)	0.39
I understood what my radiology results meant	954/1051 (90.8 %)	557/612 (91.0 %)	0.98 (0.66-1.44)	0.91	1078/1191 (90.5 %)	705/779 (90.5 %)	0.91 (0.62-1.36)	0.90

#### CTC performed when colonoscopy judged unsuitable

Of the 1191 screenees who underwent CTC after being judged unsuitable for colonoscopy, 161 underwent a subsequent colonoscopy (to investigate CTC-diagnosed abnormality). These individuals reported no significant difference in discomfort between their CTC (30/161 = 18.6 % experienced greater than expected discomfort) and colonoscopy procedures (32/161 = 19.9 %, *p* = 0.89). Similarly, there was no significant difference in rates of asking for the test to be stopped or paused (CTC: 6/161 = 3.7 %, colonoscopy: 5/161 = 3.1 %, *p* > 0.99) or post-test anorectal/abdominal pain (CTC: 21/161 = 13.0 %; colonoscopy: 24/161 = 14.3 %, *p* = 0.75).

#### CTC performed after incomplete colonoscopy

**Fig. 2:**
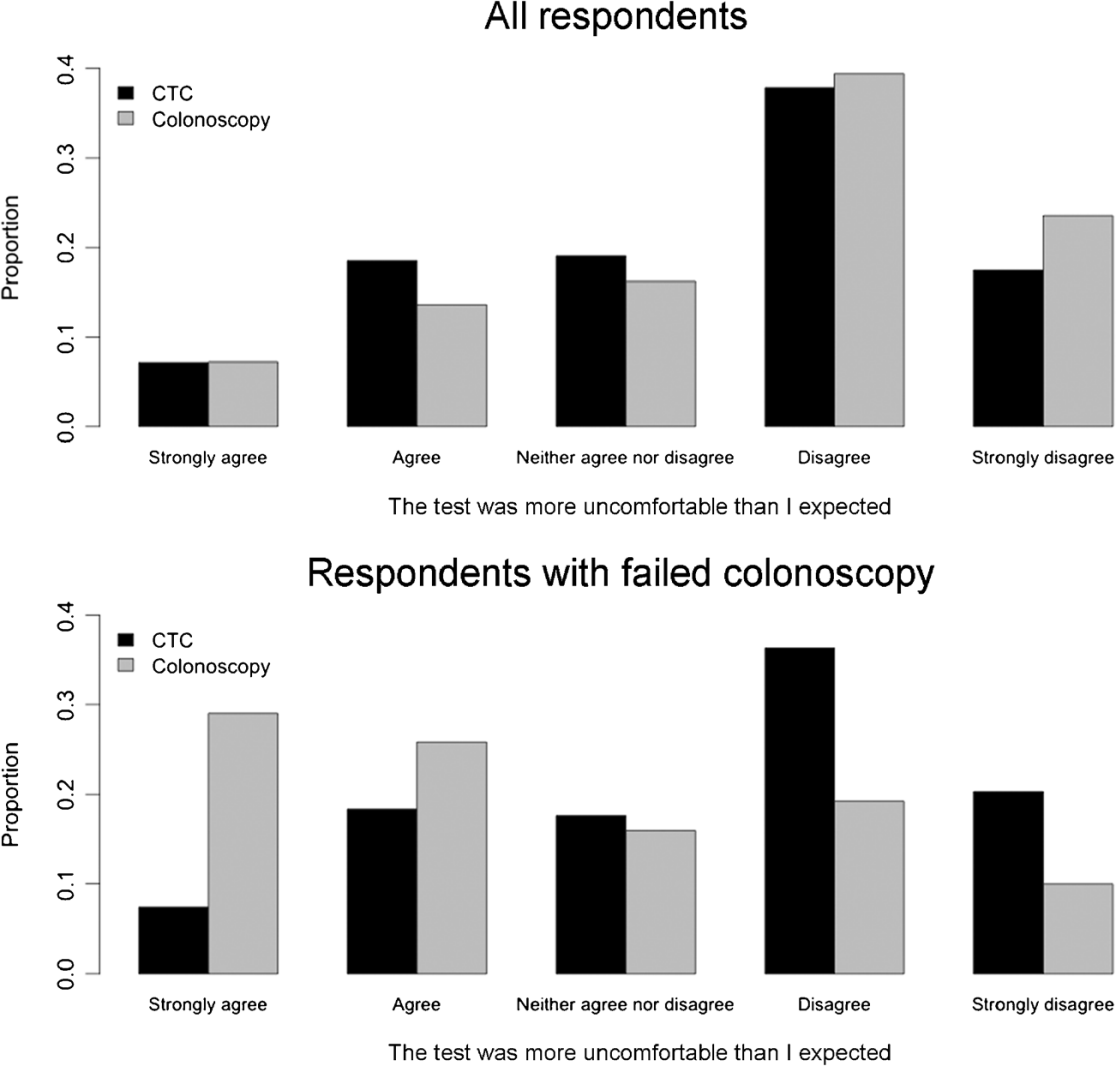
Responses to the question “The test was more uncomfortable than I expected” for all respondents (*top panel*) and those who had undergone CTC after a previous attempt at colonoscopy (*bottom panel*). Overall, respondents were more likely to agree that CTC was more uncomfortable than they had expected, whereas the opposite was true for those who had undergone CTC following colonoscopy

Screenees who had CTC following colonoscopy (using CTC as a completion test) completed 779 questionnaires. A significantly greater proportion of this subgroup found colonoscopy more uncomfortable than expected when compared with CTC (CTC: 205/779, 26.3 %; colonoscopy: 444/779, 57.0 %, *p* < 0.001). Similarly, these individuals reported more anorectal/abdominal pain after colonoscopy (187/779, 24.0 %) than after CTC (108/779, 13.9 %, *p* < 0.001, Fig. 2).

### Variation across screening centres

**Fig. 3:**
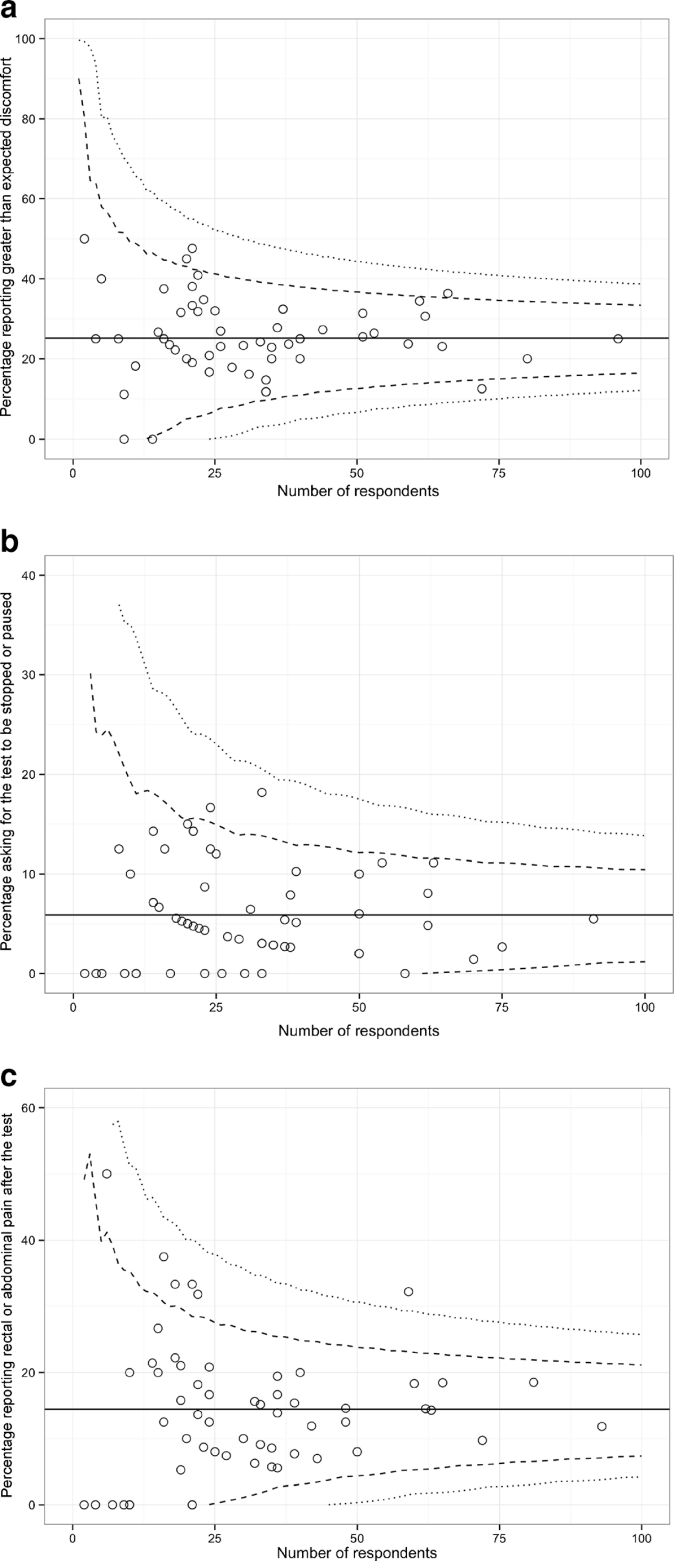
Funnel plots depicting variation between screening centres for the three “discomfort-related” measures of CTC. Each *circle* represents the response for a given screening centre, plotted against the number of respondents at that centre, for (**a**) greater than expected discomfort, (**b**) asking for the test to be stopped or paused, and (**c**) rectal or abdominal pain after the test. *Dashed* and *dotted lines* represent 95 % and 99.9 % control limits, respectively

There was moderate variation between different centres regarding the proportion of screenees reporting more than expected pain (median centre = 25.0 %, IQR = 20.0-31.8 %), anorectal or abdominal pain after the procedure (median = 13.8 %, IQR = 7.8-19.2 %), and requirement for the test to be stopped or paused (median = 5.0 %, IQR = 2.6-9.7 %). However, numbers of responses from individual centres were small, meaning that these variations largely remained within control limits (see funnel plots, Fig. 3). After adjustment for covariates, centres using reduced-laxative bowel preparation had no differences in greater than expected levels of test discomfort, post-test pain, requirement for the test to be stopped/paused or perceived clarity of the bowel preparation instructions.

### Post-procedural complications

Data regarding complications were available for 2947 individuals (2988 CTC examinations). Fifteen screenees had complications recorded, which were attributed to CTC (nine severe pain, five adverse reactions to bowel preparation or intravenous medication, and one venous thromboembolism), giving a per-test and per-screenee complication rate of 0.5 %. An additional 20 screenees experienced complications (15 severe pain, five with bleeding) as a result of further tests provoked by abnormal or incomplete CTC, giving a total complication rate (including downstream complications) of 1.2 %. No patient had perforation. A total of 66,783 colonoscopies were performed in 64,312 individuals, of whom 683 had complications, corresponding to a per-test rate of 1.0 % and a per-patient rate of 1.1 %. However, more serious complications were recorded after colonoscopy, including 34 perforations, 10 cardiac arrhythmias, and two respiratory arrests.

## Discussion

Colonoscopy and CTC are the preferred methods of pan-colonic investigation for screening and symptomatic patients. Most reports of large-scale implementation have focused on detection rates and safety [20, 26, 27], but patient experience is also an important facet. Using self-reported patient questionnaires, we documented patient experience in a national FOBt-based CRC screening programme employing CTC in accordance with international consensus [10, 11]. Overall satisfaction was high for both tests, although CTC was slightly more likely to be judged unexpectedly uncomfortable than was colonoscopy. There was no difference in the rate of intolerable symptoms requiring either test to be stopped or paused, or in anorectal/abdominal pain after the procedure. Complication rates were similar, but less serious after CTC. These data show the “real-world” acceptability between the two tests, when employed as per current practice recommendations. It is extremely reassuring that although CTC is specifically reserved for individuals who are challenging to investigate (either because colonoscopy is contraindicated or it has already failed), overall acceptability and safety of the test is high. Indeed, differences from colonoscopy (which is performed for the majority of patients, most of whom will be healthy) were small and all <5 %.

At first sight, our findings regarding patient comfort are somewhat surprising. Screenees were more likely to report that CTC was unexpectedly uncomfortable than colonoscopy, although the absolute difference was small (~5 %). This is surprising, since CTC is generally viewed as the less invasive procedure. However, the questionnaire does not record opinions regarding absolute test-related discomfort-only relative discomfort in comparison to expectations. Respondents may have had an unrealistic impression of CTC, leading to any level of discomfort being greater than expected. Conversely, expectations of colonoscopy may have been for substantial discomfort. A previous randomised trial of CTC versus colonoscopy showed that participants expected significantly greater burden from colonoscopy than from CTC [18]. We, therefore, suspect that expectations of CTC were for minimal discomfort, and even fairly mild symptoms may have breached this threshold. Additionally, those undergoing colonoscopy are frequently offered sedoanalgesia, a factor which might further mitigate any differences in test discomfort, although we found that unexpected discomfort was more common at CTC even when compared to the subgroup of individuals who did not report having sedation for their colonoscopy (Table 3). This may again be due to expectations; screenees probably expect unsedated colonoscopy to be uncomfortable, and they are generally a self-selecting, motivated, and otherwise healthy cohort [28].

Nonetheless, a similar proportion of screenees requested that CTC be stopped or paused to colonoscopy (irrespective of use of sedation), which implies similar levels of absolute test-related discomfort. One contributing factor might be inherent differences between individuals undergoing CTC and colonoscopy. For example, those undergoing CTC were older than those having colonoscopy (albeit only by approximately 6 months), and more likely to be female. Furthermore, by definition CTC was only performed when colonoscopy was incomplete or deemed unsuitable initially. Such individuals may be particularly prone to finding any colonic procedure uncomfortable. It is important to emphasise that this is the precise manner in which CTC is generally employed in FOBt-based screening programmes; therefore, our data directly reflect clinical practice. We were unable to demonstrate an effect of reduced-laxative preparation on patient comfort, perhaps because the questionnaire only records comfort during or after the CTC procedure rather than symptoms from bowel purgation itself, or underpowering.

Importantly, individuals who had colonoscopy prior to CTC (i.e. failed endoscopy) had high levels of discomfort at colonoscopy, but tolerated CTC significantly better; indeed, as well as those in whom CTC was used as a first-line test after positive FOBt. We hope that this finding will allow clinicians to reassure screenees who have had a difficult experience at colonoscopy that they are likely to find CTC tolerable.

CTC generated relatively few direct complications, as expected for such a safe procedure. While the complication rate of CTC itself was lower than for colonoscopy, this difference disappeared once considering downstream complications due to further tests precipitated by CTC. However, complications arising after CTC were not serious: No patient had perforation as a result of CTC (either directly or downstream), whereas perforation occurred at a rate of approximately 1 in 1900 following colonoscopy, comparable with existing literature [26, 29, 30].

Strengths of this study include the large sample size, high response rate (79 %), and multicenter nature with national coverage. Although missing data were noted, this was not problematic. However, our study has limitations. As noted above, the participants were not randomised, and therefore, differences may be due to variation between the tested populations rather than directly reflecting test characteristics. Arguably, this is unimportant from a clinical perspective: CTC was employed precisely as is recommended by international consensus [10, 11], meaning our results reflect clinical practice. However, we would strongly caution against using these data to argue that CTC or colonoscopy is the better-tolerated test when employed as a primary screening tool for asymptomatic subjects: CTC was reserved for those who are unsuitable for colonoscopy (roughly 60:40 split of contraindications to colonoscopy vs. incomplete colonoscopy) and was well-tolerated overall, supporting its use in this role. It is impossible to know whether or not these potentially vulnerable individuals would have found colonoscopy more or less tolerable than CTC. Our data are likely generalizable to mass screening programmes based on FOBt/FIT, but will not reflect the relative patient experience of colonoscopy or CTC when employed as first-line screening tests.

Although test complications are recorded centrally, we cannot be certain that all relevant complications were captured and uploaded successfully. Additionally, due to the large size of the dataset, most comparisons were highly statistically significant, even when absolute differences were small. It is, therefore, largely a matter of judgement whether or not the differences we report here are clinically meaningful or not.

In summary, both CTC and colonoscopy have high levels of satisfaction when used to investigate a positive FOBt result in a national CRC screening programme. Although screenees directed to CTC are a different population to those having colonoscopy, overall test tolerability was good, with only a small increase in test discomfort and no difference in post-procedural pain or complication rates. Screenees requiring CTC after incomplete colonoscopy tolerated CTC extremely well. Individuals requiring CTC may require more detailed information regarding expected procedural discomfort.
